# Transcriptional analysis of *bla*_NDM-1_ and copy number alteration under carbapenem stress

**DOI:** 10.1186/s13756-017-0183-2

**Published:** 2017-02-20

**Authors:** Deepjyoti Paul, Amitabha Bhattacharjee, Dibyojyoti Bhattacharjee, Debadatta Dhar, Anand Prakash Maurya, Atanu Chakravarty

**Affiliations:** 1 Department of Microbiology, Assam University, Silchar, India; 2 Department of Statistics, Assam University, Silchar, India; 3 Department of Microbiology, Silchar Medical College and Hospital, Silchar, India

**Keywords:** *Escherichia coli*, Plasmid stability, Transcriptional expression, Transferability

## Abstract

**Background:**

New Delhi metallo beta-lactamase is known to compromise carbapenem therapy and leading to treatment failure. However, their response to carbapenem stress is not clearly known. Here, we have investigated the transcriptional response of *bla*_NDM-1_ and plasmid copy number alteration under carbapenem exposure.

**Methods:**

Three *bla*_NDM-1_ harboring plasmids representing three incompatibility types (IncFIC, IncA/C and IncK) were inoculated in LB broth with and without imipenem, meropenem and ertapenem. After each 1 h total RNA was isolated, immediately reverse transcribed into cDNA and quantitative real time PCR was used for transcriptional expression of *bla*_NDM-1_. Horizontal transferability and stability of the plasmids encoding *bla*_NDM-1_ were also determined. Changes in copy number of *bla*_NDM-1_ harboring plasmids under the exposure of different carbapenems were determined by real time PCR. Clonal relatedness among the isolates was determined by pulsed field gel electrophoresis.

**Results:**

Under carbapenem stress over an interval of time there was a sharp variation in the transcriptional expression of *bla*_NDM-1_ although it did not follow a specific pattern. All *bla*_NDM-1_ carrying plasmids were transferable by conjugation. These plasmids were highly stable and complete loss was observed between 92^nd^ to 96^th^ serial passages when antibiotic pressure was withdrawn. High copy number of *bla*_NDM-1_ was found for IncF type plasmids compared to the other replicon types.

**Conclusion:**

This study suggests that the single dose of carbapenem pressure does not significantly influence the expression of *bla*_NDM-1_ and also focus on the stability of this gene as well as the change in copy number with respect to the incompatible type of plasmid harboring resistance determinant.

**Electronic supplementary material:**

The online version of this article (doi:10.1186/s13756-017-0183-2) contains supplementary material, which is available to authorized users.

## Background

Since the discovery of New Delhi metallo-β-lactamase (*bla*_NDM_) in 2008 from a Swedish patient of Indian origin in New-Delhi, India [1], this enzyme is known for several reasons including treatment failure, emergence of new variants and lateral transfer of the gene coding this enzyme within diverse host range of Gram negative bacilli [2, 3]. The *bla*_NDM_ is known for its ignominious nature being linked with other resistance determinants along with various mobile elements like plasmid, insertion sequences & transposons which facilitates its horizontal dissemination [2, 4]. In many studies *bla*_NDM-1_ was found to be associated with IS*Aba125* [2, 5]. However, there were also reports of other insertion elements like IS*CR1*, IS*CR16*, IS*26*, IS*1*, IS*Ec33* and IS*903* associated with this gene [5]. Additionally, the transposons Tn*3* and Tn*125* were reported to be linked with this resistance determinant and horizontal transfer of *bla*_NDM-1_ is often facilitated by plasmids of IncF, IncA/C, IncL/M, IncH, IncN and more recently by IncX type [6]. Among Enterobacteriaceae, *bla*_NDM_ was detected in *Escherichia coli* in many countries worldwide (Australia, France, Germany, Japan, UK and the USA) [7]. *E. coli* is the most common pathogen associated with nosocomial and community acquired infections and also been considered as a potent host for this resistance determinant [7]. Dissemination of *bla*_NDM-1_ through *E. coli* has become a global concern [8] and also in India there were several reports of NDM-producing *E. coli* in all parts of the country [8–14]. Treatment of infections with NDM-producers is restricted due to their multidrug resistance phenotype [15]. Several studies have highlighted the hydrolytic activity of NDM-1 to carbapenems [2, 16]. However, it is not known how bacteria harboring this resistance gene will respond when carbapenem therapy is initiated to a patient. The present study was designed to investigate the transcriptional response of *bla*_NDM-1_ in vitro under single dose carbapenem exposure, and also to investigate the transmission dynamics within clinical isolates of *Escherichia coli* in a single center study from India.

## Methods

### Bacterial strains

The NDM-1 producing *E. coli* isolates (*n* = 17) were collected from different clinical specimens (stool, *n* = 3; surgical wound, *n* = 1; urine, *n* = 3; pus, *n* = 5; throat swab, *n* = 1; ear swab, *n* = 1; endotracheal aspirates, *n* = 1; cerebrospinal fluid, *n* = 1; blood, *n* = 1) of seventeen patients who were admitted in different wards or attended to outpatient departments (OPDs) of Silchar Medical College and Hospital (Silchar, India) from March till September 2013. The isolates were identified by standard biochemical characterization and 16 s rDNA sequencing [17]. Presence of *bla*_NDM_ was determined by PCR assay using primers (NDM-F 5^/^-GGGCAGTCGCTTCCAACGGT-3^/^and NDM-R 5^/^-GTAGTGCTCAGTGTCGGCAT-3^/^ [18]. The amplified products were purified using MinElute PCR Purification Kit (Qiagen, Germany) and were sequenced.

### Transcriptional expression analysis of *bla*_NDM-1_

Transcriptional expression of *bla*_NDM-1_ in response to imipenem, meropenem and ertapenem stress was determined by inoculating the organisms harboring *bla*_NDM-1_ in Luria Bertani broth (Hi-media, Mumbai, India) with and without antibiotics. Antibiotic concentration used was 1 μg/ml. For a period of 16 h, total RNA was isolated at the interval of 1 h using Qiagen RNease Mini Kit (Qiagen, Germany), immediately reverse transcribed into cDNA by using QuantiTect^®^ reverse transcription kit (Qiagen, Germany). The cDNA was quantified by Picodrop (Pico 200, Cambridge, UK) and real time PCR was performed using Power Sybr Green Master Mix (Applied Biosystem, Warrington, UK) in Step One Plus real time detection system (Applied Biosystem, USA) using a set of primer (NDM-F 5^/^-GGGCAGTCGCTTCCAACGGT-3^/^and NDM-RT-R 5^/^-CGACCGGCAGGTTGATCTCC-3^/^). The relative expression of *bla*_NDM-1_ in each interval with and without carbapenem pressure was determined by ΔΔC_t_ method [19]. Relative quantification was done using a transformant (*E. coli* DH_5_α harboring *bla*_NDM_; P^EC-611^) grown for 4 h without any antibiotic pressure.

### Transformation and Conjugation assay

Transformation was performed by heat shock method [15] using *E. coli* DH_5_α as a recipient and the transformants were selected on Luria Bertani agar (Hi-Media, Mumbai, India) containing 0.25 μg/ml of imipenem. Conjugation experiment was carried out using *bla*_NDM-1_ harboring clinical strains as donors and a streptomycin resistant *E. coli* recipient strain B (Genei, Bangalore, India). The MIC of clinical isolates against streptomycin was pre-determined to optimize the agar for selection of transconjugants. Both the donor and recipient cells were cultured in Luria Bertani Broth (Hi-Media, Mumbai, India) till it reach an O.D. of 0.8–0.9 at A_600_. Cells were mixed at 1:5 donor-to-recipient ratios and transconjugants were selected on agar plates containing imipenem (0.25 μg/ml) and streptomycin (1000 μg/ml). The *E. coli* strain B is chromosomally resistant to streptomycin which can grow on media containing streptomycin at a concentration of 1000 μg/ml. However, the donors although resistant to aminoglycoside had the minimum inhibitory concentration ranging from 100-200 μg/ml. Therefore, selection of transformants in 1000 μg/ml rules out false selection of donor strains. The accuracy of conjugation was further cross checked by typing all the transconjugants by enterobacterial repetitive intergenic consensus PCR [20] and pulsed field gel electrophoresis using Xba1 restriction enzyme.

### Replicon typing and plasmid stability analyses

Incompatibility type of the plasmid encoding *bla*_NDM-1_ was determined by PCR based replicon typing targeting 18 different replicons viz. FIA, FIB, FIC, HI1, HI2, I1/Iγ, L/M, N, P, W, T, A/C, K, B/O, X, Y, F and FIIA as described previously [21]. Also IncX types i.e. IncX1, IncX2, IncX3 and IncX4 were also targeted [22]. Purified plasmid DNA was used as template for the reaction.

Plasmid stability analysis of parent strains and transformants was done by serial passage method for consecutive 100 days at 1:1000 dilutions without any antibiotic pressure [23]. After each passage, 1 ml of the culture was diluted in normal saline (1:1000) and 40 μl of the diluted sample was spread on to the LB agar plate. After overnight incubation, 50 colonies from the agar plates were randomly picked and subjected to phenotypic detection of MBL and further confirmed by PCR assay for the presence of *bla*_NDM-1_ using primers (NDM-F 5^/^-GGGCAGTCGCTTCCAACGGT-3^/^and NDM-R 5^/^-GTAGTGCTCAGTGTCGGCAT-3^/^.

### Copy number determination of plasmid encoding *bla*_NDM-1_

Clinical isolates of *Escherichia coli* harboring *bla*_NDM-1_ carried by plasmids of incompatibility groups IncFIC, IncA/C or IncK were selected for determining the copy number under exposure of different concentrations of carbapenem antibiotics. Single colony of each incompatibility type was inoculated into LB broth containing 0.5 μg/ml, 1 μg/ml, 2 μg/ml and 4 μg/ml of each imipenem, meropenem and ertapenem and also without any antibiotic (considered as a reference), was incubated at 37 °C for 5–6 h until the OD reached 0.9 at A_600_. Transformants with different *bla*_NDM-1_ carrying plasmid types (IncFIC, A/C & K) were used as control (without any antibiotic pressure). Plasmid DNA was extracted using QIAprep Spin Miniprep Kit (Qiagen, Germany). Quantitative Real Time PCR was performed using Step One Plus real time detection system (Applied Biosystem, USA) to estimate the relative copy number of *bla*_NDM-1_ for different concentrations of each antibiotic for three different incompatibility types. The copy number of *bla*_NDM-1_ within the wild type plasmid of different incompatibility types were also determined to know the type of Inc group where copy number of *bla*_NDM-1_ gene was maintained in high number. Quantitative real time PCR reaction was carried out using 10 μl of SYBR® Green PCR Master Mix (Applied Biosystem, Warrington, UK), 4 ng plasmid DNA as template and 3 μl of each primer (10 Picomol) in a 20 μl reaction under a reaction condition of initial denaturation at 94 °C for 5 min, 40 cycles of denaturation 94 °C for 20 s, annealing 52 °C for 40 s and extension at 72 °C for 30 s. The relative fold change was measured by ΔΔ*CT* method and Ct value of each sample was normalized against a housekeeping gene *rpsel* of *E. coli* [19].

### Antimicrobial susceptibility testing and MIC determination

Antibiotic susceptibility of *bla*_NDM-1_ harboring parent strains, transformants and transconjugants were determined by Kirby Bauer disc-diffusion method including piperacillin-tazobactam (100/10 μg), co-trimoxazole (25 μg), amikacin (30 μg), gentamicin (10 μg), ciprofloxacin (5 μg), polymyxin B (300units), netilmicin (30 μg), carbenicillin (100 μg), tigecycline(30 μg) and faropenem (5 μg) (Hi-Media, Mumbai, India). MICs of imipenem, meropenem, ertapenem, cefepime, aztreonam, gentamicin, amikacin, ciprofloxacin, piperacillin-tazobactam & polymixin-B were determined for parent strains harboring *bla*_NDM-1,_ as well as transformants and transconjugants by agar dilution method. Each stock solution for the corresponding antibiotic was made at 1 mg/ml concentration in nuclease free water and was stored at −80 °C. The quality control for stock solution was checked each time against *E. coli* ATCC 25922. The result of the susceptibility testing was interpreted as per CLSI guidelines [24]. However, for polymyxin B, faropenem and carbenicillin, the organisms were considered as non susceptible if the MIC value was higher and diameter of the zone of inhibition was lower than the values given in CLSI guidelines for respective antibiotics against *E. coli* ATCC 25922.

### Typing of *bla*_NDM-1_ harboring isolates

All *bla*_NDM-1_ harboring *E. coli* isolates were typed by pulsed field gel electrophoresis (PFGE), genomic DNA was prepared in agarose blocks and digested with the restriction enzyme Xba1 (Promega, Madison, USA) and the DNA fragments were separated with a CHEF-DR III (Bio-Rad, USA) for 24 h at 6 V/cm with a pulses at 120^0^ angle in a 10–40 s pulse time [25].

## Results

**Fig. 1:**
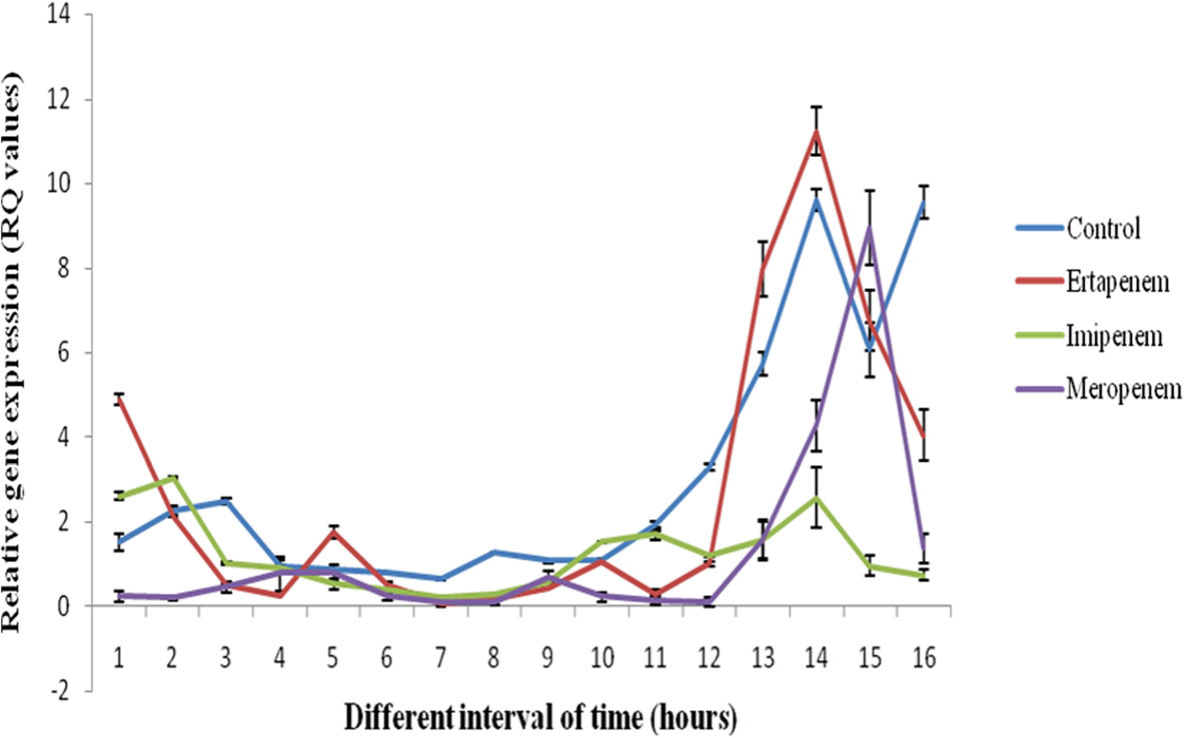
Transcriptional response of *bla*_NDM-1_ against carbapenem exposure at different time interval

During the study period (March-September), 17 isolates were obtained carrying *bla*_NDM-1_, collected from different clinical samples mostly associated with surgical wound infection from surgery ward of the hospital (Table 1). Transcriptional expression of *bla*_NDM-1_ with or without carbapenem stress is shown in Fig. 1. It was observed that at the initial stage, under meropenem pressure the transcriptional level of *bla*_NDM-1_ was low. However, there was a sharp increase from 12^th^ hour of incubation for meropenem and ertapenem (approximately 2 fold and 4 fold respectively), whereas imipenem did not cause any alteration in transcriptional response. Overall the transcriptional expression did not show any specific pattern of response.

**Table 1:** Characteristics of seventeen *Escherichia coli* isolates carrying *bla*_NDM-1_ on conjugative plasmids

Strain ID	Date of Isolation	Patient’s Sex & Age	Sample origin	Ward	*bla*_NDM-1_ positive plasmids		Pulsotypes
					Name	Inc-type	
EC51	5-3-2013	M-27 years	Stool	Medicine	P^EC-51^	FIC	P_1_
EC54	11-3-2013	M-26 years	Stool	Medicine	P^EC-54^	A/C	P_4_
EC61	11-3-2013	M-31 years	Surgical wound	Surgery	P^EC-61^	K	P_1_
EC75	13-3-2013	F-47 years	Stool	Medicine	P^EC-75^	untypeable	P_1_
EC177	7-4-2013	M-33 years	Urine	Medicine	P^EC-177^	A/C	P_3_
EC178	7-4-2013	F-12.5 years	Pus	Surgery	P^EC-178^	FIC	P_2_
EC255	13-5-2013	M-48 years	Pus	Surgery	P^EC-255^	FIC	P_2_
EC355	27-6-2013	M-10 years	Urine	Medicine	P^EC-355^	untypeable	P_2_
EC456	6-7-2013	F-25 years	Throat swab	ENT	P^EC-456^	A/C	P_5_
EC472	9-7-2013	F-62 years	Pus	Orthopedics	P^EC-472^	K	P_6_
EC477	9-7-2013	F-32 years	Ear swab	ENT	P^EC-477^	FIC	P_3_
EC489	16-7-2013	M-8.5 years	Pus	Surgery	P^EC-489^	K	P_2_
EC492	19-7-2013	M-11 years	Endotracheal aspirates	OPD	P^EC-492^	untypeable	P_6_
EC571	15-8-2013	F-21 years	Cerebrospinal fluid	Surgery	P^EC-571^	FIC	P_2_
EC611	29-8-2013	F-42 years	Blood	Surgery	P^EC-611^	A/C	P_2_
EC639	7-9-2013	M-20 years	Urine	Medicine	P^EC-639^	FIC	P_5_
EC678	16-9-2013	M-38 years	Pus	Surgery	P^EC-678^	FIC	P_4_

**Fig. 2:**
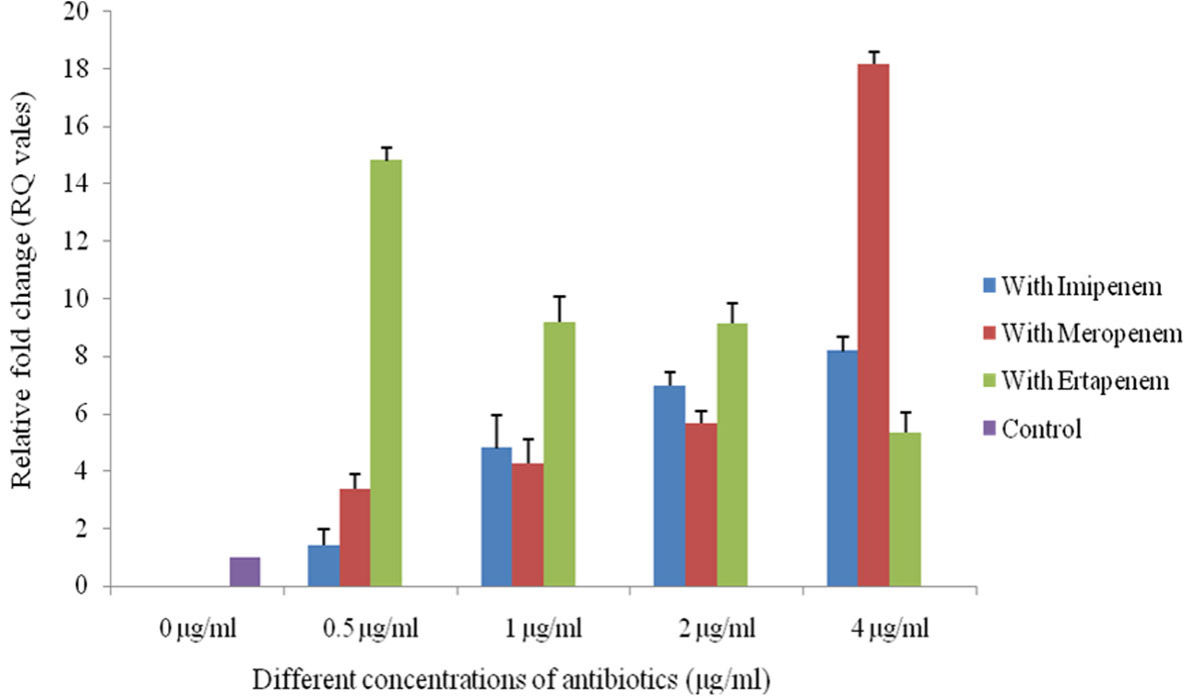
Copy number of *bla*_NDM-1_ within IncFIC plasmid. 0 μg/ml (control) = copy number of *bla*_NDM-1_ without any antibiotic pressure. 0.5, 1, 2 and 4 μg/ml = change in copy number of *bla*_NDM-1_ under 0.5, 1, 2 and 4 μg/ml exposure of imipenem (*blue bar*), meropenem (*red bar*) and ertapenem (*green bar*) pressure. The error bars represent the standard deviation of the three replicates of one sample

**Fig. 3:**
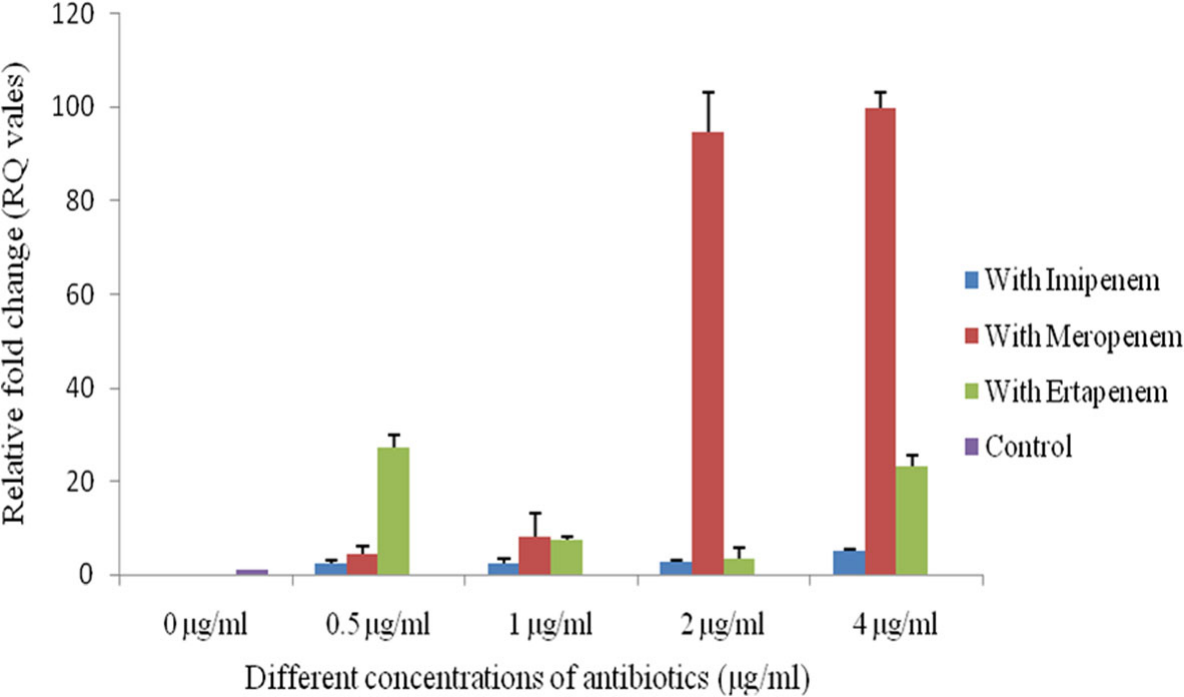
Copy Number of *bla*_NDM-1_ within IncA/C plasmid. 0 μg/ml (Control) = Copy number of *bla*_NDM-1_ without any antibiotic pressure. 0.5, 1, 2 and 4 μg/ml = Change in copy number of *bla*_NDM-1_ under 0.5, 1, 2 and 4 μg/ml exposure of imipenem (*blue bar*), meropenem (*red bar*) and ertapenem (*green bar*) pressure. The error bars represent the standard deviation of the three replicates of one sample

**Fig. 4:**
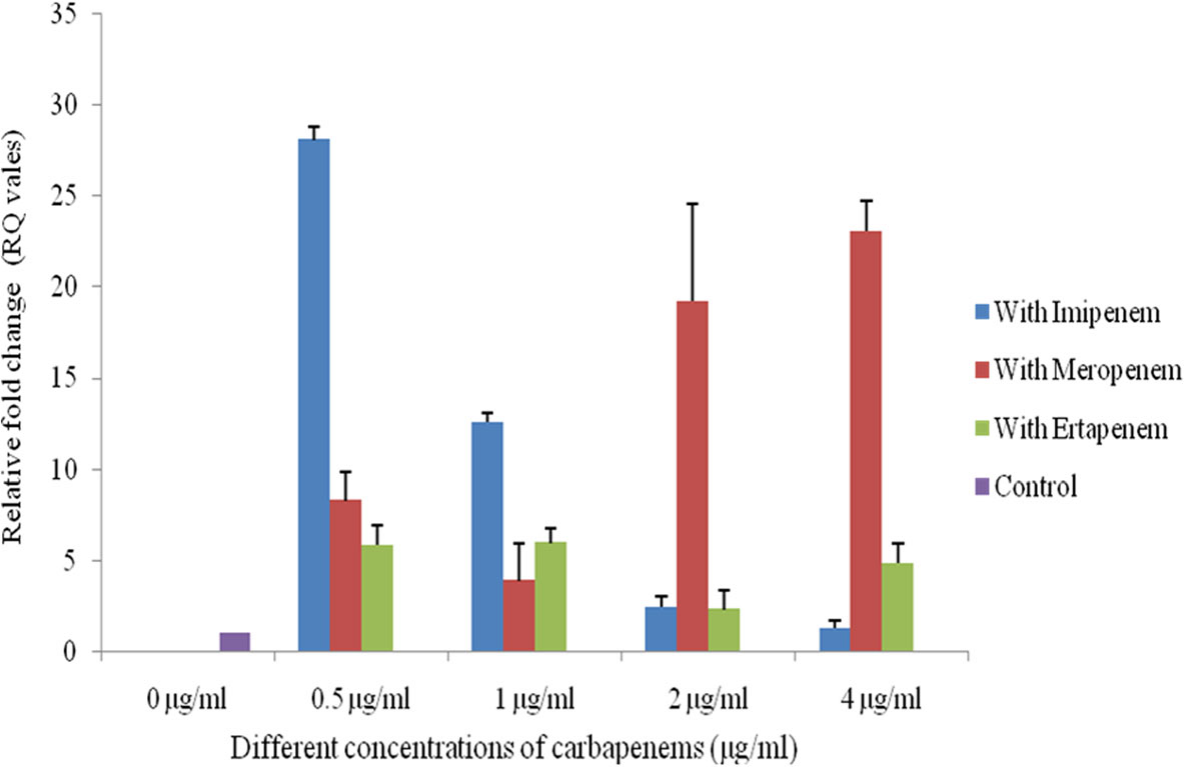
Copy Number of *bla*_NDM-1_ within IncK plasmid. 0 μg/ml (Control) = Copy number of *bla*_NDM-1_ without any antibiotic pressure. 0.5, 1, 2 and 4 μg/ml = Change in copy number of *bla*_NDM-1_ under 0.5, 1, 2 and 4 μg/ml exposure of imipenem (*blue bar*), meropenem (*red bar*) and ertapenem (*green bar*) pressure. The error bars represent the standard deviation of the three replicates of one sample

**Fig. 5:**
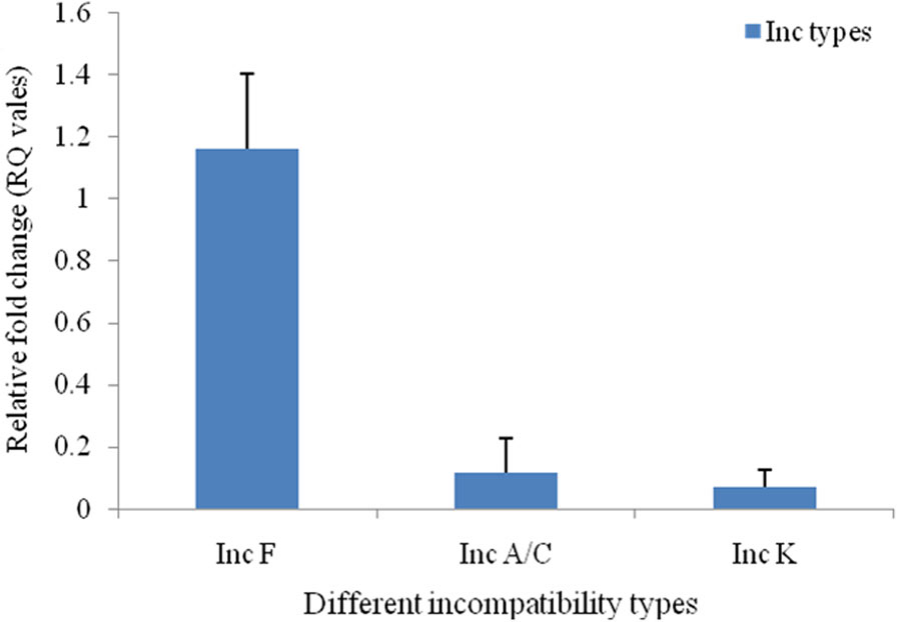
Relative copy number of IncF, A/C and K plasmid. The error bars represent the standard deviation of the three replicates of one sample

Plasmids carrying *bla*_NDM-1_ were selected in the medium containing imipenem and could be conjugatively transferred from all 17 clinical *E. coli* isolates into recipient *E. coli* strain B*.* The transformation experiment revealed that the size of the transferable plasmids was approximately of 50-60 kb. Replicon typing showed that FIC was the predominant replicon type (*n* = 7) followed by A/C (*n* = 4) and K (*n* = 3) whereas 3 isolates were untypeable (Table 1). The copy number of *bla*_NDM-1_ was found to be variable. The copy number of *bla*_NDM-1_ gene within IncFIC and IncA/C type of plasmids showed an increasing trend when increasing concentrations of imipenem and meropenem were added whereas for ertapenem, the case was reverse (Figs. 2 & 3). For IncK type plasmids, the copy number of *bla*_NDM-1_ consistently raised when meropenem concentration was increased whereas with the increasing concentration of imipenem and ertapenem, the copy number of *bla*_NDM-1_ reduced (Fig. 4). The overall copy number of F-Inc type was six fold higher compared to IncA/C and K type (Fig. 5). Complete loss of plasmids for all the isolates containing *bla*_NDM-1_ was observed between 92^nd^ to 96^th^ serial passages when antibiotic pressure was withdrawn.

Antimicrobial susceptibility result showed that the 17 *bla*_NDM-1_ harboring isolates were resistant to co-trimoxazole, ciprofloxacin, carbenicillin and faropenem whereas very few isolates were found to be susceptible to polymyxin B (*n* = 4) and tigecycline (*n* = 3) (Additional file 1: Table S1). MIC results revealed that the parent strains carrying *bla*_NDM-1_ showed MIC range above the breakpoint for all three carbapenems (≥64 μg/ml), third generation cephalosporin (≥256 μg/ml), piperacillin/tazobactam (≥32 μg/ml), polymyxin-B (≥1 μg/ml) aminoglycosides, quinolone and monobactam (≥64 μg/ml) (Additional file 1: Table S1). Transformants and transconjugants carrying *bla*_NDM-1_ were also resistant to cephalosporin, piperacillin/tazobactam, aminoglycosides, quinolone and all carbapenems (Additional file 1: Table S2). PFGE analysis revealed the presence of six different *E. coli* clones with clone 2 (pulsotype 2) as the most frequent one (*n* = 6) (Additional file 2: Figure S1). However, the replicon types of the *bla*_NDM-1_ carrying plasmids were different in this clone (IncFIC, *n* = 3; IncA/C, *n* = 1; IncK, *n* = 1; untypeable, *n* = 1).

## Discussion

Resistance to carbapenems due to the production of New Delhi metallo-β-lactamase among enterobacterial isolates has become a very common phenomenon and the expansion of *bla*_NDM-1_ among the members of Enterobacteriaceae is increasing and in consequence this resistance determinant has been reported across the globe [26]. Earlier studies demonstrated that the sub-inhibitory concentrations of antibiotics interfere the expression of the genes, colonization and motility of the cell [27]. Therefore, we have investigated the transcriptional response of NDM-1 against carbapenem antibiotics below the inhibitory concentration level. Under the pressure of imipenem, no significant change was observed in the pattern of transcriptional level for 16 h duration, which is in contrast to the previous report of Liu et al. 2012 [28], as they reported that under the pressure of imipenem *bla*_NDM-1_ gene was expressed (0.83 times higher) than that of the control. In this study, a possible down regulated expression of *bla*_NDM-1_ took place under the exposure of meropenem, however to support our data no existing literature is available till date. This study has pointed that no specific or defined transcriptional response is initiated for *bla*_NDM-1_ when carbapenem stress is created and the overall response is partially chaotic. Thus, there could be other inducing factors which trigger its response in order to synthesis this carbapenemase. The study isolates showed resistance to almost all the antibiotics especially high rate of polymyxin resistance was also observed. The emergence of different *E. coli* clones with pulsotype 2 as the most common, indicates a possible clonal spread but different replicon types within this clone are uncommon and require further detailed analyses in future studies.

Plasmids encoding *bla*_NDM-1_ gene were successfully transferred to the recipient *E. coli* strain B by conjugation indicating potential horizontal transmission through diverse incompatible plasmid types such as IncFIC, IncA/C and IncK in this hospital setting. Association of *bla*_NDM-1_ with IncK type of plasmid in the present study is not commonly reported as coexisting data recorded recent spread of *bla*_NDM-1_ in India has been associated with IncA/C type, IncF1/FII-type, or unknown types of plasmids [7]. An earlier study [29] suggested that copy number of *bla*_NDM-1_ is affected by the concentration of imipenem. In contrast we observed that plasmid copy number is not only related with high concentration of imipenem but also depends on the replicon type of the *bla*_NDM-1_ carrying plasmids. This could be supported by the high copy number of *bla*_NDM-1_ within IncF type plasmids compared to the other replicon types (e.g. IncA/C or Inc K).

## Conclusion

The expression of *bla*_NDM-1_ could predict the bacterial response in different time interval when a single carbapenem exposure is applied. Additionally, this study could underscore that irrespective of plasmid types, *bla*_NDM-1_ is highly stable within a host of clinical origin. However, it was also evident from this study that different Inc types of plasmids have a specific pattern in copy number alteration under concentration gradient carbapenem stress. Thus, the study came up with epidemiological knowledge of a stable *bla*_NDM-1_ mediated carbapenem resistance in *E. coli* and further investigation is required to evaluate the risk for their dissemination in health care systems in this geographical part of the world.

## Additional files

Additional file 1:
**Table S1.** Antimicrobial profile of *bla*_NDM-1_ harboring *Escherichia coli* isolates SXT: Trimethoprim/sulfamethoxazole, TGC: Tigecycline, FAR: Faropenem, CIP: Ciprofloxacin, CAR: Carbenicillin, PMB: Polymixin B, AMK: Amikacin, GEN: Gentamicin, NET: Netilmicin, TZP: Piperacillin/tazobactum, IPM: Imipenem, ETP: Ertapenem, MEM: Meropenem, FEP: Cefepime, ATM: Aztreonam. **Table S2.** Susceptibility pattern of transformants and transconjugants carrying *bla*_NDM-1_ IPM: Imipenem, ETP: Ertapenem, MEM: Meropenem, FEP: Cefepime, ATM: Aztreonam, GEN: Gentamicin, AMK: Amikacin, CIP: Ciprofloxacin, TZP: Piperacillin/tazobactum, PMB: Polymixin B T.F (transformants) = recipient *E. coli* DH5α carrying plasmid encoding *bla*_NDM-1_ T.C (transconjugants) = recipient *E. coli* strain B strain carrying plasmid encoding *bla*_NDM-1_. (DOC 139 kb)
Additional file 2: Figure S1. PFGE analysis showed six pulsotypes of *Escherichia coli* harboring *bla*_NDM-1_ Lane 1: High range ladder; Lane 2: EC51; Lane 3: EC54; Lane 4: EC61; Lane 5: EC75; Lane 6: EC177; Lane 7: EC178; Lane 8: EC255; Lane 9: EC355; Lane 10: EC456; Lane 11: EC472; Lane 12: EC477; Lane 13: EC489; Lane 14: EC492; Lane 15: EC571; Lane 16: EC611; Lane 17: EC639; Lane 18: EC678. (TIF 4160 kb)

## Abbreviation

*bla*_NDM-1_: New-Delhi metallo β-lactamase
cDNA: Complementary deoxyribonucleic acid
CLSI: Clinical laboratory standard institute.
C_t_: Threshold cycle
DNA: Deoxyribonucleic acid
Inc type: Incompatibility type
LB: Luria bertani
MIC: Minimum inhibitory concentration
O.D: Optical density
OPD: Outpatient department
PCR: Polymerase chain reaction
PFGE: Pulsed field gel electrophoresis
RNA: Ribonucleic acid
