# Supplementary material for: Transcriptional analysis of blaNDM-1 and copy number alteration under carbapenem stress
Source: Antimicrob Resist Infect Control. 2017 Feb 20;6:26. doi: 10.1186/s13756-017-0183-2 (PMC5319162; doi:10.1186/s13756-017-0183-2)
Supplement: Additional file 1: — Table S1. Antimicrobial profile of bla NDM-1 harboring Escherichia coli isolates SXT: Trimethoprim/sulfamethoxazole, TGC: Tigecycline, FAR: Faropenem, CIP: Ciprofloxacin, CAR: Carbenicillin, PMB: Polymixin B, AMK: Amikacin, GEN: Gentamicin, NET: Netilmicin, TZP: Piperacillin/tazobactum, IPM: Imipenem, ETP: Ertapenem, MEM: Meropenem, FEP: Cefepime, ATM: Aztreonam. Table S2. Susceptibility pattern of transformants and transconjugants carrying bla NDM-1 IPM: Imipenem, ETP: Ertapenem, MEM: Meropenem, FEP: Cefepime, ATM: Aztreonam, GEN: Gentamicin, AMK: Amikacin, CIP: Ciprofloxacin, TZP: Piperacillin/tazobactum, PMB: Polymixin B T.F (transformants) = recipient E. coli DH5α carrying plasmid encoding bla NDM-1 T.C (transconjugants) = recipient E. coli strain B strain carrying plasmid encoding bla NDM-1. (DOC 139 kb) [file 13756_2017_183_MOESM1_ESM.doc]

**Table S1: Antimicrobial profile of *bla*_NDM-1_ harboring *Escherichia coli* isolates**

| **Serial number** | **Strain ID** | **Resistance profile** | **MIC (mg/L)** | | | | | | | | | |
| --- | --- | --- | --- | --- | --- | --- | --- | --- | --- | --- | --- | --- |
|  |  |  | **IPM** | **ETP** | **MEM** | **FEP** | **ATM** | **GEN** | **AMK** | **CIP** | **PIT** | **PB** |
| 1 | EC 51 | SXT,TGC, FAR, CIP,CAR,PMB,AMK,GEN,NET | >256 | >256 | 256 | >256 | 256 | 64 | 64 | 256 | 32 | 128 |
| 2 | EC 54 | TZP,SXT,AMK,GEN,CIP,PMB,NET,CAR,FAR,TGC | 256 | >256 | >256 | >256 | >256 | 256 | >256 | 256 | >256 | >256 |
| 3 | EC 61 | TZP,SXT,AMK,GEN,CIP,TGC,NET,CAR,FAR | >256 | 128 | 256 | >256 | 128 | 128 | >256 | >256 | >256 | 4 |
| 4 | EC 75 | TZP,SXT,AMK,GEN,CIP,PMB,NET,CAR,FAR,TGC | 128 | 256 | 256 | 256 | >256 | >256 | >256 | 128 | >256 | 256 |
| 5 | EC 177 | TZP,SXT,AMK,GEN,CIP,PMB,TGC,NET,CAR,FAR | >256 | >256 | >256 | >256 | 256 | 256 | >256 | >256 | 256 | >256 |
| 6 | EC 178 | TZP,SXT,AMK,GEN,CIP,NET,CAR,FAR,PMB | 256 | 256 | 256 | >256 | 128 | >256 | 256 | 256 | 256 | 128 |
| 7 | EC 255 | SXT,AMK,GEN,CIP,TGC,NET,CAR,FAR,PMB,TZP | >256 | >256 | >256 | >256 | >256 | >256 | >256 | >256 | >256 | 256 |
| 8 | EC 355 | TZP,SXT,AMK,GEN,CIP,PMB,NET,CAR,FAR,TGC | 256 | 256 | 64 | 256 | 64 | 128 | 256 | 128 | 128 | 64 |
| 9 | EC 456 | SXT,TGC,AMK,GEN,FAR,CAR,CIP,PMB,TZP | >256 | 128 | 256 | >256 | 256 | >256 | >256 | >256 | 256 | 256 |
| 10 | EC 472 | SXT,CAR,AMK,GEN,FAR,CIP,TZP,NET,TGC | 256 | >256 | >256 | >256 | 256 | >256 | 256 | >256 | >256 | 1 |
| 11 | EC 477 | TZP,SXT,AMK,GEN,CIP,NET,CAR,FAR,PMB | 128 | >256 | 128 | >256 | >256 | >256 | >256 | 64 | >256 | 256 |
| 12 | EC 489 | SXT,CIP,PMB,CAR,FAR,AMK,GEN,TZP,NET | 256 | >256 | 256 | >256 | 256 | >256 | 256 | >256 | 256 | 128 |
| 13 | EC-492 | TZP,SXT,AMK,GEN,CIP,PMB,TGC,NET,CAR,FAR | >256 | 256 | >256 | >256 | 256 | 256 | 256 | 128 | >256 | >256 |
| 14 | EC-571 | SXT,CAR,FAR,AMK,GEN,CIP,NET,TZP,TGC | 256 | >256 | 256 | 256 | >256 | >256 | >256 | >256 | 256 | 2 |
| 15 | EC-611 | SXT,CAR,FAR,GEN, CIP,TGC, AMK,GEN,NET | 128 | 128 | 128 | >256 | 128 | 128 | >256 | 64 | 128 | 2 |
| 16 | EC-639 | TZP,SXT,AMK,GEN,CIP,TGC,CAR,FAR,PMB | >256 | 256 | >256 | >256 | 256 | >256 | >256 | >256 | >256 | >256 |
| 17 | EC-678 | TZP,SXT,AMK,GEN,CIP,PMB,NET,CAR,FAR,TGC | 256 | 256 | 128 | >256 | 256 | 256 | >256 | >256 | >256 | 128 |

**Table S2: Susceptibility pattern of transformants and transconjugants carrying *bla***NDM-1

| Strain  ID | **MIC(mg/L)** | | | | | | | | | | | | | | | | | | | | | | | | | | | |
| --- | --- | --- | --- | --- | --- | --- | --- | --- | --- | --- | --- | --- | --- | --- | --- | --- | --- | --- | --- | --- | --- | --- | --- | --- | --- | --- | --- | --- |
|  | **IPM** | | | **ETP** | | | **MEM** | | | **FEP** | | | **ATM** | | | **GEN** | | | **AMK** | | | **CIP** | | **PIT** | | **PB** | | |
|  | **T.F** | **T.C** | | **T.F** | **T.C** | | **T.F** | | **T.C** | **T.F** | **T.C** | | **T.F** | | **T.C** | **T.F** | **T.C** | | **T.F** | | **T.C** | **T.F** | **T.C** | **T.F** | **T.C** | **T.F** | | **T.C** |
| EC 51 | 8 | 8 | | 4 | 4 | | 8 | 4 | | 16 | 16 | | 4 | | 2 | 32 | 32 | | 64 | | 64 | 16 | 16 | 64 | 64 | 0.5 | | 0.5 |
| EC 54 | 16 | 8 | | 16 | 16 | | 32 | 16 | | 32 | 32 | | 4 | | 4 | 64 | 64 | | 64 | | 64 | 8 | 16 | 64 | 64 | 1 | | 1 |
| EC 61 | 4 | 4 | | 8 | 8 | | 8 | 4 | | 16 | 16 | | 2 | | 2 | 16 | 16 | | 32 | | 32 | 32 | 32 | 64 | 32 | 0.25 | | 0.5 |
| EC 75 | 16 | 16 | | 16 | 16 | | 32 | 16 | | 64 | 32 | | 8 | | 4 | 32 | 16 | | 128 | | 64 | 8 | 16 | 32 | 16 | 0.125 | | 0.25 |
| EC 177 | 8 | 8 | | 8 | 16 | | 16 | 16 | | 16 | 16 | | 8 | | 8 | 16 | 16 | | 64 | | 64 | 16 | 16 | 16 | 16 | 0.125 | | 0.125 |
| EC 178 | 32 | 32 | | 16 | 32 | | 32 | 32 | | 64 | 32 | | 4 | | 2 | 64 | 64 | | 128 | | 128 | 32 | 32 | 128 | 256 | 1 | | 1 |
| EC 255 | 16 | 16 | | 32 | 32 | | 16 | 16 | | 32 | 32 | | 8 | | 16 | 32 | 64 | | 64 | | 64 | 64 | 128 | 256 | 256 | 1 | | 1 |
| EC 355 | 64 | 64 | | 64 | 32 | | 64 | 64 | | 64 | 64 | | 16 | | 16 | 128 | 128 | | 128 | | 128 | 64 | 64 | 128 | 128 | 1 | | 1 |
| EC 456 | 64 | 32 | | 32 | 32 | | 32 | 64 | | 32 | 64 | | 8 | | 8 | 64 | 64 | | 64 | | 64 | 32 | 32 | 64 | 64 | 0.5 | | 0.25 |
| EC 472 | 32 | 32 | | 64 | 64 | | 32 | 64 | | 64 | 32 | | 8 | | 4 | 32 | 32 | | 64 | | 32 | 64 | 64 | 128 | 128 | 0.25 | | 0.25 |
| EC 477 | 8 | 4 | | 8 | 8 | | 16 | 8 | | 16 | 8 | | 4 | | 2 | 32 | 32 | | 32 | | 32 | 16 | 32 | 64 | 64 | 1 | | 0.5 |
| EC 489 | 8 | 8 | | 4 | 8 | | 4 | 8 | | 8 | 16 | | 2 | | 2 | 64 | 64 | | 128 | | 64 | 32 | 16 | 32 | 64 | 0.5 | | 0.5 |
| EC-492 | 16 | 16 | | 32 | 16 | | 16 | 8 | | 32 | 32 | | 2 | | 2 | 128 | 128 | | 128 | | 128 | 32 | 32 | 128 | 64 | 0.25 | | 0.25 |
| EC-571 | 4 | 4 | | 8 | 8 | | 8 | 4 | | 16 | 16 | | 2 | | 1 | 64 | 64 | | 64 | | 64 | 64 | 64 | 64 | 32 | 1 | | 0.5 |
| EC-611 | 8 | 4 | | 4 | 4 | | 4 | 8 | | 8 | 8 | | 4 | | 2 | 64 | 64 | | 32 | | 32 | 8 | 8 | 16 | 16 | 0.5 | | 0.5 |
| EC-639 | 4 | | 4 | 8 | | 4 | 8 | 8 | | 16 | | 16 | 2 | 2 | | 32 | | 32 | 64 | 64 | | 128 | 256 | 16 | 16 | 0.25 | 0.5 | |
| EC-678 | 8 | | 16 | 16 | | 16 | 32 | 16 | | 32 | | 16 | 4 | 4 | | 64 | | 32 | 32 | 64 | | 64 | 64 | 32 | 32 | 0.25 | 0.25 | |
| *E. coli* DH_5_α | 0.06 | | | 0.012 | | | 0.06 | | | 0.06 | | | 0.12 | | | 0.125 | | | 0.06 | | | 0.06 | | 0.12 | | 0.006 | | |
| *E. coli* strain B | 0.06 | | | 0.06 | | | 0.06 | | | 0.12 | | | 0.125 | | | 0.06 | | | 0.125 | | | 0.12 | | 0.125 | | 0.006 | | |
