# Supplementary figures and images for: Transcriptional analysis of blaNDM-1 and copy number alteration under carbapenem stress
Source: Antimicrob Resist Infect Control. 2017 Feb 20;6:26. doi: 10.1186/s13756-017-0183-2 (PMC5319162; doi:10.1186/s13756-017-0183-2)

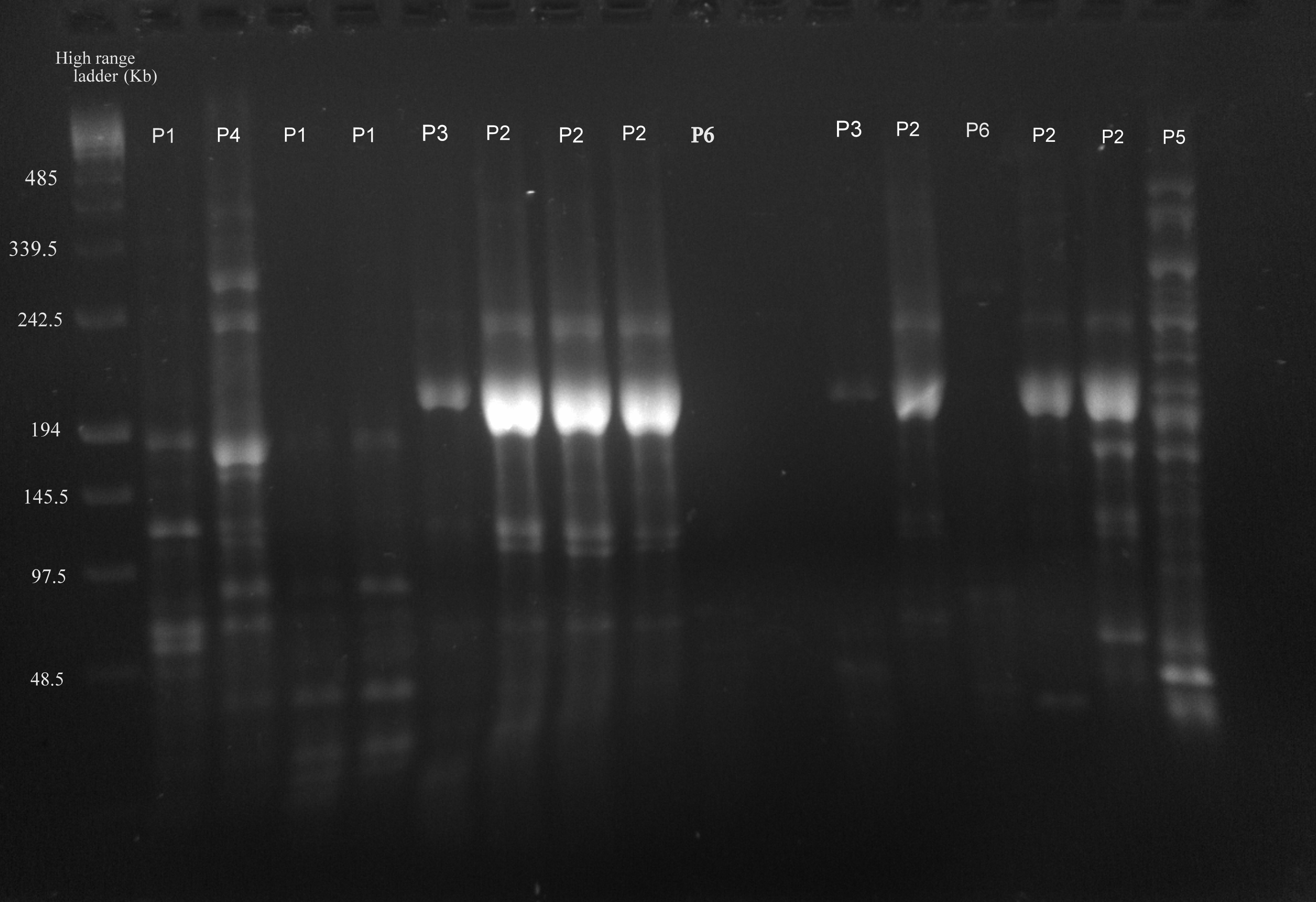

Supplement: Additional file 2: Figure S1. — PFGE analysis showed six pulsotypes of Escherichia coli harboring bla NDM-1 Lane 1: High range ladder; Lane 2: EC51; Lane 3: EC54; Lane 4: EC61; Lane 5: EC75; Lane 6: EC177; Lane 7: EC178; Lane 8: EC255; Lane 9: EC355; Lane 10: EC456; Lane 11: EC472; Lane 12: EC477; Lane 13: EC489; Lane 14: EC492; Lane 15: EC571; Lane 16: EC611; Lane 17: EC639; Lane 18: EC678. (TIF 4160 kb) [file 13756_2017_183_MOESM2_ESM.tif]
